# Candidate microRNAs as biomarkers of thyroid carcinoma: a systematic review, meta‐analysis, and experimental validation

**DOI:** 10.1002/cam4.811

**Published:** 2016-07-27

**Authors:** Yiren Hu, Hui Wang, Ende Chen, Zhifeng Xu, Bi Chen, Guowen Lu

**Affiliations:** ^1^ Department of General SurgeryThe Third Clinical College of Wenzhou Medical University, Wenzhou People's HospitalWenzhouChina; ^2^ Department of General SurgeryNingbo Yinzhou People's HospitalNingboChina; ^3^ Department of Oncological SurgeryWenzhou People's Hospital, The Third Clinical College of Wenzhou Medical UniversityWenzhouChina; ^4^ Department of Thyroid and breast mininally invasive surgeryNingbo Yinzhou People's HospitalNingboChina

**Keywords:** Biomarker, meta‐analysis, miRNA, thyroid cancer

## Abstract

Thyroid cancer is one of the most common carcinomas of the endocrine system with an increasing incidence. A growing number of studies have focused on the diagnostic and prognostic values of dysregulated microRNAs (miRNAs) in thyroid carcinoma. However, differences in the measurement platforms, variations in lab protocols, and small sample sizes can make gene profiling data incomparable. A meta‐review of the published studies that compared miRNA expression data of thyroid carcinoma and paired normal tissues was performed to identify potential miRNA biomarkers of thyroid carcinoma with the vote‐counting strategy. Two hundred and thirty‐six aberrantly expressed miRNAs were reported in 19 microRNA expression profiling studies. Among them, 138 miRNAs were reported in at least two studies. We also provided a meta‐signature of differentially expressed miRNAs between individual histological types of thyroid carcinoma and normal tissues. The experimental validation with qRT‐PCR analysis verified that the profiles identified with the meta‐review approach could effectively discriminate papillary thyroid carcinoma tissues from paired noncancer tissues. The meta‐review of miRNA expression profiling studies of thyroid carcinoma would provide information on candidate miRNAs that could potentially be used as biomarkers in thyroid carcinoma.

## Introduction

Thyroid carcinoma represents the most frequent carcinoma of the endocrine system 1. Most thyroid cancers originate from thyroid follicular cells (>90%) and can be subdivided into well‐differentiated papillary thyroid carcinoma (PTC) and follicular thyroid carcinoma (FTC), while only less than 5% originate from C‐cell, often referred to as medullary thyroid carcinoma (MTC) 2. The most common follicular tumor is benign hyperplastic adenoma, whereas PTC represents the most frequent thyroid carcinoma (about 90%). PTC and FTC may progress to poorly differentiated carcinoma or can fully lose differentiation to give rise to anaplastic thyroid carcinoma (ATC) 3.

A large number of studies have been performed to screen candidate biomarkers for thyroid carcinoma. Quite a lot of molecular variations have been identified in thyroid carcinoma tissues 4, 5, 6. miRNAs are a class of noncoding RNAs, which are between 19 to 25 nucleotides in length. They have been demonstrated to be potential early cancer detection biomarkers, prognostic indicators, and therapeutic targets 7, 8. miRNAs exert function via binding to the complementary sites in the 3′ untranslated region of target mRNAs to promote target gene mRNA degradation or inhibit translation 9. Studies have showed that miRNAs are involved in a wide array of cellular processes, including proliferation, apoptosis, metastasis, and cellular differentiation 10, 11, 12.

High‐throughput technologies have been employed to screen the expression of miRNAs across normal and cancer tissues. These studies could result in hundreds or thousands of aberrantly expressed miRNAs, while only a small portion of them may be of actual clinical utility. Furthermore, with respect to the identified meta‐signature of miRNAs, great inconsistency existed among different studies. Finding a meaningful combination from different datasets is usually not an easy job. Differences in measurement platforms, variations in experiment protocols, limited numbers of samples studied, and low numbers of aberrantly expressed miRNAs in comparison to relatively large total numbers of miRNAs, may render miRNA expressions levels uninterpretable. Therefore, it might be better to analyze datasets separately and thereafter aggregate the miRNA list. Such a strategy has been a success in finding human gene coexpression networks 13 and in defining more accurate list of cancer‐related genes 14, 15 and miRNAs 8, 16, 17.

We could use the meta‐review approach, which combines the miRNAs expression profiling results to increase the statistical power for working out the inconsistency or discrepancies. In this study, a meta‐review of published miRNAs expression profiles across normal and thyroid cancer tissues was performed. Then we used the well‐known meta‐analysis method, the vote‐counting strategy 14, 15, and ranked the miRNAs based on the number of profiling studies consistently reporting this miRNA, total sample size and average fold change. The meta‐analysis was first carried out in all histological types of thyroid carcinoma (PTC, follicular thyroid carcinoma (FTC), medullary thyroid carcinoma (MTC), and ATC). Then, a meta‐analysis was performed in four subtypes of thyroid carcinoma, respectively.

## Materials and Methods

### Selection of studies and datasets

A search for thyroid carcinoma miRNA expression profiling studies was performed in PubMed using the following keywords: “miRNA” OR “microRNA” OR “miR”, “thyroid carcinoma”, “profiling” OR “microarray”. The latest search was performed on 25 February 2016. Titles and abstracts of the obtained articles were screened, and full texts of the articles of interest were further evaluated. Original articles published in English that analyzed miRNA expression between thyroid carcinoma and noncancerous thyroid tissue in humans were included. Exclusion criteria: (1) articles published in non‐English language; (2) case reports or review articles; (3) studies with the method of qRT‐PCR for initial screening; (4) studies using serum or plasma of thyroid cancer patients; (5) studies not using the method of miRNA microarray or sequencing platform for initial screening; (6) profiling of histological subtypes other than the predetermined histological subtypes (PTC, FTC, MC and ATC); (7) studies not including noncancerous normal tissues; (8) detailed information of platforms were not available; (9) profiling of benign thyroid tumor samples; (10) profiling across metastatic and nonmetastatic, recurrent and nonrecurrent, aggressive and nonaggressive thyroid carcinoma tissues; and (11) profiling studies not across malignant thyroid carcinomas and normal thyroid tissues.

### Data extraction

The two authors (YH and YW) performed the online search, evaluation and extraction of data utilizing the standard protocol independently, with the discrepancies resolved by discussion with the third author (EC). The information listed below were retrieved from the full texts and supplemental materials: author, time of publication, country of subjects, year of sample analysis, clinical characteristics of the enrolled thyroid carcinoma patients, characteristics of measurement platforms, list of dysregulated miRNA features, cut‐off criteria of statistically differentially expressed miRNAs, and fold changes. miRNA annotation were standardized to miRBase Release 21.

### Ranking

MiRNAs were ranked according to the order of importance below: (1) number of studies reporting the same miRNAs with a consistent direction of aberration; (2) total number of profiling samples in the same direction of change; and (3) average fold changes for the same miRNAs reported consistently. We consider total sample size to be more important than average fold change as fold changes were not available in many studies. Average fold change was calculated with the method of weighted mean, mean = (x_1_f_1 _+ x_2_f_2 _+ … x_k_f_k_)/(f_1 _+ …f_k_), x_k_ stands for fold change of a single study, f_k_ stands for sample size. In studies where fold changes were not reported, the 2^−ΔΔ^Ct method was used to determine fold change between two groups. The relative expression of miRNA was calculated with reference to expression of house‐keeping genes and expressed as fold changes.

### Sample collection

Twenty‐five PTC samples and paired noncancer thyroid tissue samples were collected between October 2014 and May 2016 after radical surgical section at the Department of Thyroid and Breast Mininally Invasive Surgery, Ningbo Yinzhou People's Hospital (Ningbo, China). The diagnoses were finally made by skilled pathologists. Once the surgical specimens were removed, research personnel instantly transferred the PTC tissues to the lab. Pathology faculty evaluated the specimen grossly and selected the thyroid tissues that most was likely to be cancerous. Matched noncancer thyroid tissues were isolated at least 2 cm away from the tumor border and were shown to be free of tumor cells by microscopy. Each tissue samples were frozen in liquid nitrogen immediately and stored at −80°C in a refrigerator for RNA isolation.

### RNA extraction and quantitative real‐time PCR (qRT‐PCR)

Total RNA from tissues was extracted using Trizol reagent (Invitrogen, Carlsbad, CA) according to the manufacturer's protocol. Total RNA was reverse transcribed into cDNA using the Primer‐Script™ one‐step RT‐PCR kit (TaKaRa, Dalian, China) in total volume of 25 *μ*L including 1 *μ*g total RNA, 1 *μ*mol/L reverse transcription primer, 0.5 nmol/L dNTPs, 8 U M‐MLV reverse transcriptase, and 1 U RNA inhibitor by reverse transcription PCR with the following cycling parameters: 16°C for 30 min, 42°C for 30 min, and 85°C for 5 min. Real‐time PCR was performed using the SYBR Select Master Mix (Applied Biosystems, Carlsbad, CA cat: 4472908) in a final volume of 15 *μ*L with 1 *μ*L cDNA, 0.7 mmol/L forward and reverse primer, and 7.5 *μ*L SYBR Green Mater Mix. The optimum thermal cycling parameters were as follows: 95°C for 10 min, 40 cycles of 95°C for 15 sec, 60°C for 1 min, 95°C for 15 sec, 60°C for 30 sec, and 95°C for 15 sec. Real‐time PCR was performed on ABI 7300 system (Applied Biosystems) following the manufacturer's instructions. Each individual sample, with no template control, was run in triplicate, and the average critical threshold cycle (Ct) was calculated. The relative expression of miRNA was calculated with reference to expression of U6 and expressed as ratios. The 2^−ΔΔ^Ct method was used to determine fold change between two groups. The primer sequences used in this study were as the follows: U6, 5′‐CTCGCTTCGGCAGCACA‐3′ (forward),

5′‐AACGCTTCACGAATTTGCGT‐3′ (reverse); miR‐221‐5p, 5′‐ACACTCCAGCTGGGAGCTACATTGTCTGCTGG‐3′ (forward), 5′‐CTCAACTGGTGTCGTGGA‐3′ (reverse); miR‐222‐5p, 5′‐CCCTCAGTGGCTCAGTAG‐3′ (forward), 5′‐CCACCAGAGACCCAGTAG‐3′ (reverse); miR‐34a‐5p, 5′‐GGTGTGGGCTGGCAGTGTCTT‐3′ (forward), 5′‐CCAGTGCAGGGTCCGAGGTAT‐3′ (reverse); miR‐146b‐5p, 5′‐TTTATTTATTTTGGGAACGGGAGAC‐3′(forward), 5′‐GACCTTAACATTAATATTATAACACTACCG‐3′ (reverse); miR‐21‐5p, 5′‐ACACTCCAGCTGGGTAGCTTATCAGACTGA‐3′ (forward), 5′‐TGGTGTCGTGGAGTCG‐3′ (reverse); miR‐31‐5p, 5′‐ACGCGGCAAGATGCTGGCA‐3′ (forward), 5′‐CAGTGCTGGGTCCGAGTGA‐3′ (reverse); miR‐181‐5p, 5′‐GGTTGCTTCAGTGAACATTCAACGC‐3′ (forward), 5′‐GTTAGCTATAGGGTACAATCAACGGTC‐3′ (reverse); miR‐138‐5p, 5′‐TGAGAAGCACGACCTTCATGT‐3′ (forward), 5′‐GGAACCCCTATGACCTCTTCA‐3′ (reverse).

### Statistical analysis

The statistical analysis were performed utilizing SAS 9.2 software (SAS Institute Inc. NC, USA). Data are presented as means ± standard deviation. Student's *t*‐test was utilized for comparison between two independent groups. A *P < *0.05 (two‐sided) was considered to be statistically significant.

## Results

**Figure 1:**
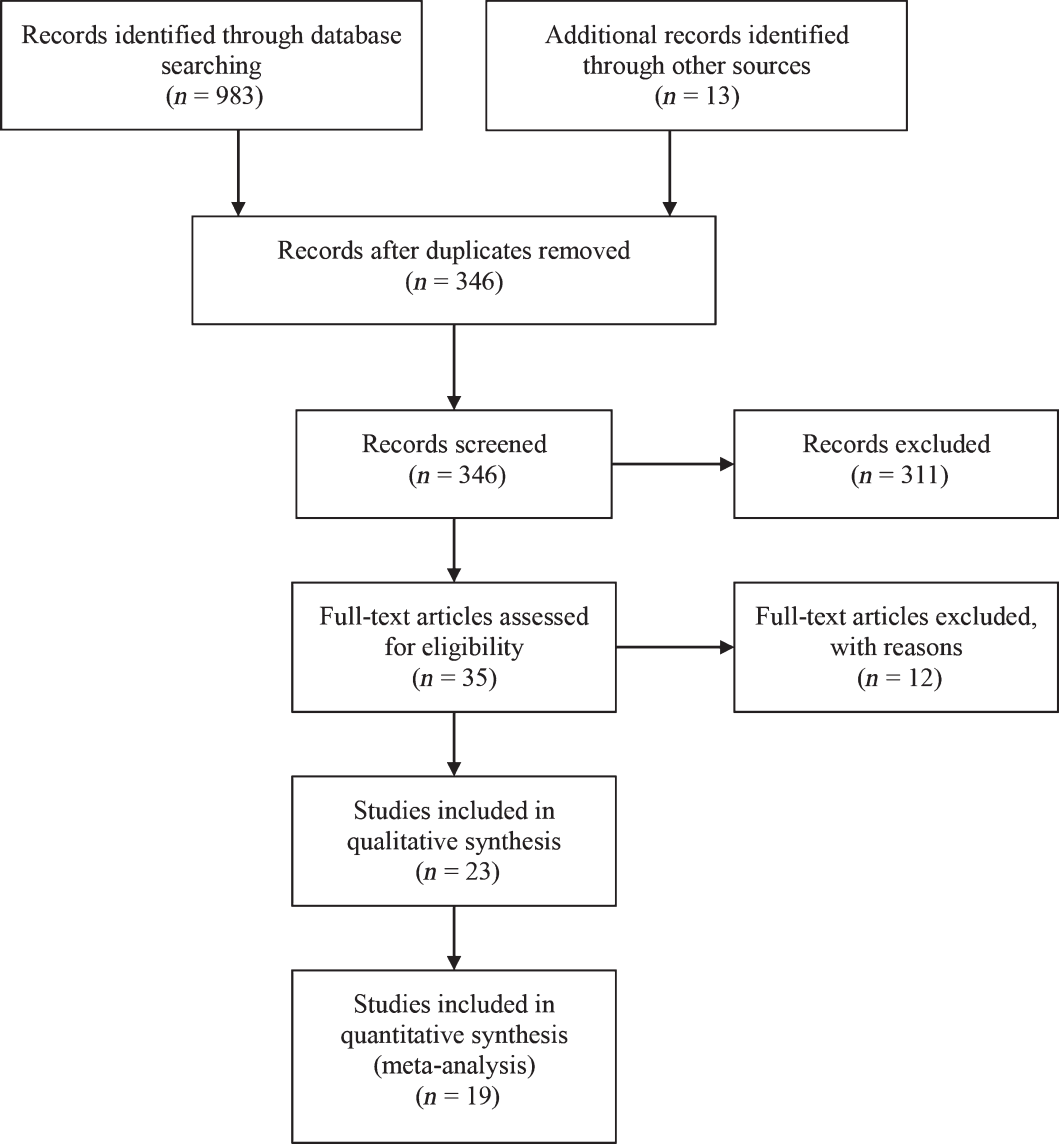
Flowchart of the study selection. Only original experimental articles that were published in English and that analyzed the differences in miRNA expression between thyroid cancer tissues and normal tissues in humans were included. Articles were excluded if the studies did not use a miRNA microarray platform.

In whole, 983 relevant studies were indexed in PubMed. According to the inclusion criteria, 23 independent studies were included 18, 19, 20, 21, 22, 23, 24, 25, 26, 27, 28, 29, 30, 31, 32, 33, 34, 35, 36, 37, 38, 39, 40. However, four articles were excluded as the aberrantly expressed miRNA lists were unavailable 37, 38, 39, 40. The flowchart used in our study is shown in Figure 1. A brief description of the included 19 studies 18, 19, 20, 21, 22, 23, 24, 25, 26, 27, 28, 29, 30, 31, 32, 33, 34, 35, 36, 37 are provided in Table 1. For studies covering different histological types of thyroid carcinoma, we considered the profiling study of one single histological type as an individual study. In our study, we found that the reference 19 covered profiling study of FTC and PTC, reference 30 covered profiling study of PTC, FTC, MTC, and ATC.

**Table 1:** Nineteen microarray‐based human thyroid cancer miRNA expression profiling studies

First author (reference)	Year	Region	Platform	Total miRNA	No.of samples (cancer/normal)
Jacques 18	2013	France	GPL7683 platform (Agilent Technologies)	866	PTC:4(2/2)
Mancikova 19	2015	Spain	Genome Analyzer IIx	>808	FTC:40(23/17)
Wang 20	2013	China	Agilent Human miRNA Microarray (8*60K,v16.0;Agilent Technologies)	1205	PTC:8(6/2)
Tetzlaff 21	2007	USA	GPL3699 (Agilent Technologies)	754	PTC:20(10/10)
Zhang 22	2013	China	*μ*Paraflo^®^ Microfluidics Biochip (LC Sciences)	NR	PTC:6(3/3)
Braun 23	2010	Germany	PIQORTM miRXplore microarrays	773	ATC:6(3/3)
Peng 24	2014	China	miRCURY^™^ LNA chip (v.16.0)	NR	PTC:8(4/4)
Kitano 25	2011	USA	miRCURY LNA array version 11.0 (Exiqon)	1263	47(26/21);PTC:14,FTC:12
Swierniak 26	2013	Poland	Custom miRNA microarray chip (OSU‐CCC version 2.0)	>427	PTC:28(14/14)
Pallante 27	2006	France	miRNA microarray chip (KCI version 1.0)	368	PTC:40(30/10)
Dettmer 28	2013	USA	TaqMan human microarray assays (Applied Biosystems)	381	FTC:31(21/10)
Vriens 29	2012	USA	miRCURY LNA (Exiqon)	850	NR
Nikiforova 30	2008	USA	TaqMan human microarray assays (Applied Biosystems)	158	PTC: 23(18/5);FTC:14(9/5);MTC:7(2/5);ATC:9(4/5)
Yip 31	2011	USA	FlexmiR^™^ human microRNA pool, version 8 (Exiqon)	319	PTC:10(6/4)
Hudson 32	2013	USA	TaqMan OpenArray MicroRNA Panel (Life Technologies)	754	MTC:20(15/5)
Visone 33	2007	Italy	miRNA microarray chip (KCI version 1.0)	248	ATC:20(10/10)
Riesco‐Eizaguirre 34	2015	Spain	Genome Analyzer IIx Platform (Illumina^®^)	NR	PTC:20(10/10)
He 35	2005	USA	New custom miRNA microarray chip (OSU‐CCC version 2.0)	460	PTC:30(15/15)
Wojtas 36	2014	Poland	Illumina miRNA Bead Array V2	NR	FTC:20(10/10)

PTC, papillary thyroid carcinoma; NR, not reported.

The number of thyroid cancer patients measured in the 19 reports ranged from 2 to 30. These studies used various kinds of microarray platforms, and the number of miRNAs assayed ranged from 158 to 1205 (mean 778; data were missing in four studies 22, 24, 34, 36). Among them, three studies 18, 19, 23 presented the whole list of aberrantly expressed miRNAs in the supplemental materials, whereas the other studies provided a part of the profiling data. Thus, we directly contacted the corresponding authors and obtained the whole data lists from corresponding authors of seven studies 20, 21, 25, 28, 29, 30, 32. The aggregated dataset included a total of 241 tumor samples and 170 noncancerous tissue samples.

In total, 19 studies reported 486 aberrantly expressed miRNAs across thyroid carcinomas and paired normal tissues. Among them, 273 were reported to be upregulated and 213 downregulated; 138 were reported in more than one study; 90 (65.22%) miRNAs were consistently reported (Tables 2 and 3) and 48 (34.78%) were reported with an inconsistent direction (Table 4). Among the consistently reported 90 miRNAs, 37 were upregulated (Table 2) and 53 were downregulated (Table 3). In the group of consistently reported microRNAs, miR‐221‐5p and hsa‐miR‐222‐5p was reported to be upregulated in 16 studies followed by miR‐146b‐5p upregulated in eleven studies. miR‐138‐5p and miR‐486‐5p were found to be downregulated in eight studies. We also provided a meta‐signature of differentially expressed miRNAs between individual histological type of thyroid carcinoma tissues and normal tissues (Tables 5, 6, 7, 8, 9, 10, 11, 12, 13, 14). PTC: Tables 5, 6, 7, FTC: Tables 8, 9, 10, MTC: Table 11, ATC: Tables 12, 13, 14. According to the results from our meta‐analysis, the top lists varied among the studies.

**Table 2:** Upregulated miRNAs (*n* = 37) in at least two expression profiling studies

miRNA name	Studies with the same direction (reference)	No. of tissue samples tested	Mean fold change	Mean rank
hsa‐miR‐221‐5p	19, 20, 21, 22, 23, 25, 26, 27, 28, 30, 30, 30, 31, 35	297	8.63	3.71
hsa‐miR‐222‐5p	20, 21, 22, 23, 25, 26, 27, 28, 30, 30, 30, 31, 33, 35	277	8.02	3.71
hsa‐miR‐146b‐5p	19, 20, 22, 24, 25, 26, 28, 30, 31, 34, 30	222	30.4	2.82
hsa‐miR‐34a‐5p	18, 19, 19, 21, 22, 27, 34, 35	200	4.67	17.25
hsa‐miR‐183‐5p	18, 19, 19, 26, 28, 34, 36	183	5.68	16.43
hsa‐miR‐182‐5p	19, 19, 22, 26, 28, 30, 34	175	4.35	20.29
hsa‐miR‐181b‐5p	19, 20, 27, 30, 30, 30, 21	139	7.59	19.29
hsa‐miR‐21‐5p	19, 20, 21, 22, 23, 34, 35	130	4.01	7.86
has‐miR‐31‐5p	20, 21, 22, 30, 31, 34	78	5.72	5.67
hsa‐miR‐146b‐3p	19, 26, 28, 34, 36	139	29.39	3.2
hsa‐miR‐96‐5p	19, 19, 20, 28, 30	129	5.72	19.2
hsa‐miR‐221‐3p	19, 19, 24, 34, 36	128	6.42	22.2
hsa‐miR‐187‐3p	19, 26, 30, 30, 30	99	28.38	15.2
miR‐213	21, 27, 35	90	2.08	7.33
hsa‐miR‐21‐3p	19, 26, 34	88	3.88	12
hsa‐miR‐214‐5p	19, 19, 30	87	12	35
hsa‐miR‐183‐3p	19, 24, 36	68	4.67	26.33
hsa‐miR‐222‐3p	19, 24, 34	68	8.02	7.33
hsa‐miR‐181a‐2‐3p	19, 22, 34	66	2.74	37.67
hsa‐miR‐375	22, 32, 34	46	12.45	3
hsa‐miR‐3613‐5p	19, 19	80	2.95	61.5
hsa‐miR‐744	25, 26	75	1.35	15
hsa‐miR‐449a	19, 28	71	4.55	42
miR‐220	27, 35	70	3.18	4.5
hsa‐miR‐147b	19, 26	68	4.52	11
hsa‐miR‐891a	19, 26	68	24.69	21.5
hsa‐miR‐32‐5p	24, 25	55	10.87	7
hsa‐miR‐526b‐3p	19, 23	46	8.91	36.5
hsa‐miR‐371a‐3p	19, 23	46	6.59	37
miR‐181c	22, 27	46	2.01	8.5
hsa‐miR‐340‐3p	19, 18	44	6.08	55
hsa‐miR‐551b	20, 26	36	18.42	2.5
hsa‐miR‐135b‐5p	20, 26	36	1.97	12.5
hsa‐miR‐10a	32, 30	27	63.6	3.5
miR‐223	21, 23	26	3.44	6.5
miR‐137	30, 30	14	90.65	3.5
hsa‐miR‐10b‐5p	18, 24	12	8.92	10.5

**Table 3:** Downregulated miRNAs (*n* = 53) reported in at least two expression profiling studies

miRNA name	Studies with the same direction (reference)	No. of tissue samples tested	Mean fold change	Mean rank
hsa‐miR‐138‐5p	19, 20, 23, 28, 29, 31, 34	112 + NR	4.48	8
hsa‐miR‐486‐5p	19, 19, 22, 23, 28, 34, 36	154	3.77	13
hsa‐miR‐151b	19, 19, 23, 27, 33	146	5.07	11.6
hsa‐miR‐30a‐5p	19, 21, 23, 25, 33	133	4.54	14.4
hsa‐miR‐30a‐3p	19, 23, 24, 32, 34	94	3.68	14.4
hsa‐miR‐451a	19, 19, 28, 34	128	4.9	7.5
hsa‐miR‐486‐3p	19, 19, 28, 36	128	4.27	10.25
hsa‐miR‐138‐1‐3p	19, 28, 33, 35	118	3.03	7
hsa‐miR‐7‐5p	19, 20, 23, 25	101	5.1	8
hsa‐miR‐126‐3p	19, 22, 23, 25	99	3.34	15.5
hsa‐miR‐204‐5p	19, 23, 28, 34	94	6.24	14.5
hsa‐miR‐100–5p	23, 28, 33, 34	54 + NR	3.28	17.5
hsa‐miR‐193a‐3p	19, 19, 25	127	2.23	29.67
hsa‐miR‐144‐5p	19, 25, 28	115	3.19	12
hsa‐let‐7 g‐3p	19, 23, 25	93	3.18	24.67
hsa‐miR‐345‐5p	19, 21, 35	90	2.16	10.33
hsa‐miR‐455–3p	28, 28, 32	79	2.32	7.33
miR‐30c	21, 23, 25	73	4.13	11
hsa‐mir‐26a‐1	23, 33, 35	56	4.69	5.67
has‐miR‐99a‐5p	23, 33, 34	46	4.01	8.33
hsa‐miR‐130a‐3p	22, 23, 28	40	2.99	17.33
hsa‐miR‐451b	19, 25	87	3.01	18
hsa‐miR‐3687	19, 19	80	11.35	10.5
hsa‐miR‐4532	19, 19	80	8.2	7
hsa‐miR‐133b	19, 19	80	3.05	29
hsa‐miR‐320c	19, 19	80	2.95	29
hsa‐miR‐320b	19, 19	80	3.3	27
hsa‐miR‐139‐5p	19, 19	80	3.4	29.5
hsa‐miR‐378d	19, 19	80	2.65	35.5
hsa‐miR‐3676‐3p	19, 19	80	3.2	29.5
hsa‐miR‐326	19, 19	80	2.45	40.5
hsa‐miR‐324‐3p	19, 19	80	2.5	37.5
hsa‐miR‐18b‐5p	19, 19	80	5.85	11.5
hsa‐miR‐378c	19, 19	80	4.4	16.5
hsa‐miR‐3609	19, 19	80	4.4	18
hsa‐miR‐6087	19, 19	80	4.65	16.5
hsa‐miR‐9‐3p	35, 36	50	2.47	5.5
hsa‐miR‐1249	19, 28	68	2.67	26
hsa‐miR‐1179	19, 28	68	7.97	3.5
hsa‐miR‐652‐3p	19, 28	68	2.52	22.5
hsa‐miR‐218‐5p	19, 21	60	2.25	26.5
hsa‐miR‐574–3p	28, 28	59	1.47	16
miR‐101	23, 25	53	3.9	15
miR‐26b	23, 25	53	3.75	17
miR‐30e‐5p	23, 25	53	3.31	20
miR‐335	22, 25	53	2.55	5
hsa‐miR‐20b‐5p	28, 32	48	2.5	8
hsa‐miR‐152	19, 23	46	3.38	34.5
miR‐15b	23, 27	46	2.78	22
hsa‐let‐7d‐5p	23, 28	34	5.82	13.5
mir‐1	31, 36	30	2.62	5.5
miR‐19b	21, 23	26	2.69	23.5
let‐7c	23, 33	26	3.64	21.5

**Table 4:** Differentially expressed miRNAs (*n* = 48) with an inconsistent direction between two studies

miRNA name	Direction of expression	Studies with the same direction	No. of tissue samples tested	Mean fold change	Mean rank
hsa‐miR‐224‐5p	↑	19, 21, 27, 30, 30, 30, 30	138	12.53	9
	↓	33	20	2.74	6
hsa‐miR‐155‐5p	↑	30, 31, 34, 35, 30, 30	91	6.58	7.33
	↓	19	40	2.6	44
hsa‐let‐7e‐5p	↑	19, 19, 18, 34	104	2.29	39.5
	↓	23	6	5	31
hsa‐miR‐199b‐5p	↑	24	8	11.48	6
	↓	19, 23, 34, 36	86	6.51	17.5
hsa‐miR‐145–5p	↑	18	4	2.72	12
	↓	21, 23, 25, 26, 33	74	2.88	13.8
hsa‐miR‐125a‐5p	↑	19, 27, 28	111	4.74	10.67
	↓	23, 33	26	3.98	16.5
hsa‐miR‐181a‐3p	↑	19, 21, 27	100	2.12	39.67
	↓	23	6	3.85	39
let‐7f‐1	↑	33	20	1.25	4
	↓	23, 25, 27	93	2.35	17.67
hsa‐miR‐195–5p	↑	18	4	1.96	22
	↓	23, 25, 26	81	4.15	15.67
hsa‐miR‐199a‐3p	↑	24	8	29.27	2
	↓	19, 23, 28	77	7.03	5.67
hsa‐miR‐29c‐5p	↑	18, 19, 35	74	2.92	38
	↓	23	6	7.14	18
hsa‐miR‐30b‐3p	↑	19	40	3.5	96
	↓	23, 25	73	5.28	13
hsa‐miR‐205‐5p	↑	19, 30, 30	61	33.07	5
	↓	36	20	3.67	4
hsa‐miR‐199a‐5p	↑	24	8	3.95	9
	↓	19, 20, 23, 28	46 + NR	4.12	15.75
miR‐125b‐1	↑	27	40	1.66	10
	↓	23, 29, 33	26 + NR	4.54	13.67
hsa‐miR‐99b‐5p	↑	19.JH	60	2.3	58.5
	↓	23, 33	26	2.1	37
hsa‐miR‐9‐5p	↑	30	7	42.1	9
	↓	19, 36	60	4.18	18
hsa‐miR‐9‐5p	↑	30	7	42.1	9
	↓	19, 36	60	4.18	18
hsa‐miR‐143‐5p	↑	18	4	7.66	5
	↓	23, 25	53	4.73	10
miR‐200a	↑	27, 30	50	4.06	7.5
	↓	23	6	6.67	19
hsa‐miR‐148b‐3p	↑	19	40	2.1	112
	↓	23, 27	46	2.03	30
hsa‐miR‐130b‐5p	↑	19, 23	46	4.65	45.5
	↓	21, 31	30	2.37	5
hsa‐let‐7a‐2‐3p	↑	33	20	1.25	3
	↓	19, 23	46	6.86	23
hsa‐miR‐30d‐3p	↑	19	40	6.6	77
	↓	23, 33	26	6.8	6
hsa‐miR‐199b‐3p	↑	18, 24	12	15.72	10.5
	↓	19	40	6.4	10
hsa‐miR‐203a	↑	24, 26	36	7.46	9
	↓	33	20	1.42	18
hsa‐miR‐29b	↑	35	30	1.7	14
	↓	23, 33	26	2.63	28
hsa‐miR‐374a	↑	18	4	2.69	13
	↓	25	47	1.98	4
hsa‐miR‐22‐5p	↑	18	4	4.71	9
	↓	25	47	1.24	20
hsa‐miR‐874	↑	28	31	2.08	9
	↓	19	40	2.2	39
miR‐125b‐2	↑	27	40	1.56	11
	↓	33	20	3.13	4
hsa‐miR‐136‐5p	↑	24	8	5.46	8
	↓	19	40	3.3	32
hsa‐miR‐514a‐3p	↑	19	40	4.4	16
	↓	24	8	7.14	2
miR‐154	↑	30	7	32.3	10
	↓	19	40	5.1	17
hsa‐miR‐150‐3p	↑	23	6	5.71	3
	↓	19	40	5.1	18
hsa‐miR‐142‐5p	↑	23	6	4.71	6
	↓	19	40	4.4	24
hsa‐miR‐149‐5p	↑	19	40	27.5	44
	↓	29	NR	16.67	1
hsa‐mir‐29a‐2	↑	35	30	1.7	13
	↓	23	6	2.13	62
hsa‐miR‐17–3p	↑	26	28	1.35	19
	↓	23	6	3.7	40
miR‐107	↑	36	20	3.22	2
	↓	23	6	3.03	52
hsa‐miR‐34b‐5p	↑	18	4	5.66	8
	↓	31	10	2	4
hsa‐miR‐150‐5p	↑	23	6	2.74	18
	↓	19, 20	8	6.1	4.5
miR‐149‐3p	↑	23	6	3.31	12
	↓	24	8	2	12
hsa‐miR‐923	↑	23	6	4.49	7
	↓	18	4	4.28	2
hsa‐miR‐494	↑	23	6	3.8	10
	↓	18	4	2.94	3
hsa‐miR‐106b	↑	18	4	1.89	23
	↓	23	6	3.13	49
hsa‐miR‐497	↑	18	4	1.66	26
	↓	23	6	2.86	53
hsa‐mir‐23b	↑	18	4	2.55	14
	↓	23	6	2.33	61

**Table 5:** Upregulated miRNAs (*n* = 31) in at least two expression profiling studies of papillary thyroid carcinoma

miRNA name	Studies with the same direction (reference)	No. of tissue samples tested	Mean fold change	Mean rank
hsa‐miR‐221‐5p	19, 20, 21, 22, 26, 27, 30, 31, 35	205	10.18	2.78
hsa‐miR‐222–5p	20, 21, 22, 26, 27, 30, 31, 35	165	9.93	3.38
hsa‐miR‐34a‐5p	19, 18, 34, 21, 22, 35, 27	120	5.1	7.17
hsa‐miR‐146b‐5p	20, 22, 24, 26, 30, 31, 34	103	31.59	1.86
hsa‐miR‐21‐5p	19, 20, 21, 22, 34, 35	124	4.18	6.67
has‐miR‐31‐5p	20, 21, 22, 30, 31, 34	87	5.72	5.67
hsa‐miR‐181b‐5p	20, 21, 27, 30, 35	121	4.75	6.4
hsa‐miR‐224‐5p	19, 21, 27, 30	123	3.57	11
hsa‐miR‐182‐5p	19, 22, 26, 34	94	2.37	14.25
hsa‐miR‐183‐5p	18, 19, 26, 34	92	3.9	17
hsa‐miR‐155‐5p	30, 31, 34, 35	83	5.2	7.75
hsa‐miR‐213	21, 27, 35	90	2.08	7.33
hsa‐miR‐21‐3p	19, 26, 34	88	3.88	12
hsa‐miR‐221‐3p	19, 24, 34	68	8.55	7
hsa‐let‐7e‐5p	18, 19, 34	64	2.15	19.33
hsa‐miR‐125a‐5p	19, 27	80	2.4	15.5
miR‐220	27, 35	70	3.18	4.5
hsa‐miR‐147b	19, 26	68	4.52	11
hsa‐miR‐96‐5p	19, 20	48	5.99	7.5
miR‐181a	21, 27	60	1.84	8.5
hsa‐miR‐187‐3p	26, 30	51	39.4	3
hsa‐miR‐146b‐3p	26, 34	48	21.9	1.5
miR‐181c	22, 27	46	2.01	8.5
hsa‐miR‐551b	20, 26	36	18.42	2.5
hsa‐miR‐135b‐5p	20, 26	36	1.97	12.5
hsa‐miR‐203a	24, 26	36	7.46	9
hsa‐miR‐29c	18, 35	34	1.97	15.5
hsa‐miR‐222‐3p	24, 34	28	5.74	7.5
hsa‐miR‐375	22, 34	26	12.45	4
hsa‐miR‐181a‐2‐3p	22, 34	26	2.21	10
hsa‐miR‐10b‐5p	18, 24	12	8.92	10.5

**Table 6:** Downregulated miRNAs (*n* = 14) reported in at least two expression profiling studies of papillary thyroid carcinoma

miRNA name	Studies with the same direction (reference)	No. of tissue samples tested	Mean fold change	Mean rank
hsa‐miR‐138‐5p	19, 20, 26, 31, 34	106	3.49	8
hsa‐miR‐486‐5p	19, 22, 26, 34	94	3.44	6.75
hsa‐miR‐138‐1‐3p	19, 26, 35	98	3.27	6.67
hsa‐miR‐345‐5p	19, 21, 35	90	2.16	10.33
hsa‐miR‐451a	19, 26, 34	88	4.06	7.33
hsa‐miR‐204‐5p	19, 26, 34	88	7.12	5
hsa‐miR‐30a‐3p	19, 24, 34	68	2.95	14.33
hsa‐miR‐486‐3p	19, 26	68	4.18	10.5
hsa‐miR‐1179	19, 26	68	7.97	3.5
hsa‐miR‐652‐3p	19, 26	68	2.52	22.5
hsa‐miR‐30a‐5p	19, 21	60	1.95	23.5
hsa‐miR‐100–5p	26, 34	48	1.89	12
hsa‐miR‐130a‐3p	22, 26	34	3.09	13.5
hsa‐miR‐130b‐5p	21, 31	30	2.37	5

**Table 7:** Differentially expressed miRNAs (*n* = 5) with an inconsistent direction between two studies of papillary thyroid carcinoma

miRNA name	Direction of expression	Studies with the same direction	No. of tissue samples tested	Mean fold change	Mean rank
hsa‐miR‐145–5p	↑	18	4	2.72	12
	↓	21, 26	48	2	10.5
hsa‐miR‐514a‐3p	↑	19	40	4.4	16
	↓	24	8	7.14	2
hsa‐miR‐205‐5p	↑	30	23	6.8	9
	↓	36	20	3.67	4
hsa‐miR‐199b‐5p	↑	24	8	11.48	6
	↓	34	20	2.5	8
hsa‐miR‐34b‐5p	↑	18	4	5.66	8
	↓	31	10	2	4

**Table 8:** Upregulated miRNAs (*n* = 12) in at least two expression profiling studies of follicular thyroid carcinoma

miRNA name	Studies with the same direction (reference)	No. of tissue samples tested	Mean fold change	Mean rank
hsa‐miR‐183‐5p	19, 28, 36	91	8.05	21.33
hsa‐miR‐182‐5p	19, 28, 30	91	6.99	28.33
hsa‐miR‐146b‐3p	19, 28, 36	91	34.38	4.33
hsa‐miR‐96‐5p	19, 28, 30	85	5.54	27
hsa‐miR‐146b‐5p	19, 28, 30	85	36.08	8.67
hsa‐miR‐449a	19, 28	71	4.35	20.29
hsa‐miR‐183‐3p	19, 36	60	5.99	36.5
hsa‐miR‐221‐3p	19, 36	60	3.23	45
hsa‐miR‐187‐3p	19, 30	54	8.97	33
hsa‐miR‐181b‐5p	19, 30	54	8.15	52.5
hsa‐miR‐221‐5p	28, 30	45	5.14	6.5
hsa‐miR‐222–5p	28, 30	45	5.64	5.5

**Table 9:** Downregulated miRNAs (*n* = 7) reported in at least two expression profiling studies of follicular thyroid carcinoma

miRNA name	Studies with the same direction (reference)	No. of tissue samples tested	Mean fold change	Mean rank
hsa‐miR‐199a‐3p	19, 28	71	5.55	6
hsa‐miR‐199a‐5p	19, 28	71	4.1	14
hsa‐miR‐486‐5p	19, 36	60	4.64	9
hsa‐miR‐486‐3p	19, 36	60	4.36	10
hsa‐miR‐199b‐5p	19, 36	60	10.45	1.5
hsa‐miR‐9‐5p	19, 36	60	4.18	18
hsa‐miR‐9‐5p	19, 36	60	4.18	18

**Table 10:** Differentially expressed miRNAs (*n* = 1) with an inconsistent direction between two studies of follicular thyroid carcinoma

miRNA name	Direction of expression	Studies with the same direction	No. of tissue samples tested	Mean fold change	Mean rank
hsa‐miR‐155‐5p	↑	MN	14	5.5	6
	↓	VM	40	2.6	44

**Table 11:** Upregulated miRNAs (*n* = 1) in at least two expression profiling studies of medullary thyroid carcinoma

miRNA name	Studies with the same direction	No. of tissue samples tested	Mean fold change	Mean rank
hsa‐miR‐10a	30, 32	27	63.6	3.5

**Table 12:** Upregulated miRNAs (*n* = 1) in at least two expression profiling studies of anaplastic thyroid carcinoma

miRNA name	Studies with the same direction (reference)	No. of tissue samples tested	Mean fold change	Mean rank
hsa‐miR‐222–5p	23, 30, 33	35	6.25	4

**Table 13:** Downregulated miRNAs (*n* = 9) reported in at least two expression profiling studies of anaplastic thyroid carcinoma

miRNA name	Studies with the same direction (reference)	No. of tissue samples tested	Mean fold change	Mean rank
hsa‐miR‐30a‐5p	23, 33	26	8.67	3.5
has‐miR‐99a‐5p	23, 33	26	5.43	9.5
hsa‐mir‐26a‐1	23, 33	26	6.21	6
let‐7c	23, 33	26	3.64	21.5
miR‐30d	23, 33	26	6.8	6
miR‐125a	23, 33	26	3.98	16.5
miR‐125b‐1	23, 33	26	4.04	18.5
miR‐29b	23, 33	26	2.63	28
miR‐99b	23, 33	26	2.1	37

**Table 14:** Differentially expressed miRNAs (*n* = 3) with an inconsistent direction between two studies of anaplastic thyroid carcinoma

miRNA name	Direction of expression	Studies with the same direction	No. of tissue samples tested	Mean fold change	Mean rank
hsa‐miR‐224‐5p	↑	30	9	12	8
	↓	33	20	2.74	6
hsa‐let‐7a‐2‐3p	↑	33	20	1.25	3
	↓	23	6	11.11	3
let‐7f‐1	↑	33	20	1.25	3
	↓	23	6	4.35	34

We validated the expression of the eight most consistently reported miRNAs (miR‐221‐5p, miR‐222‐5p, miR‐34a‐5p, miR‐146b‐5p, miR‐21‐5p, miR‐31‐5p, miR‐181‐5p, and miR‐138‐5p) in PTC using qRT‐PCR analysis. The pathological characteristics of the 25 PTC patients were presented in Table 15. The results demonstrated that the expression levels of miR‐221‐5p, miR‐222‐5p, miR‐34a‐5p, miR‐146b‐5p, miR‐21‐5p, and miR‐31‐5p were upregulated, while the expression levels of miR‐181‐5p and miR‐138‐5p were downregulated in the PTC tissues, compared with paired normal thyroid tissues (all *P *<* *0.05) (Table 16).

**Table 15:** Clinicopathological characteristics of 25 papillary thyroid carcinoma (PTC) patients

ID	Sex	Age of onset	Histological diagnosis	TNM
1	M	67	PTC	T1N1M1
2	F	66	PTC	T3N1M1
4	F	49	PTC	T3N0M1
5	F	56	PTC	T1N0M0
6	F	36	PTC	T2N1M0
7	F	41	PTC	T2N0M0
8	F	54	PTC	T2N0M0
9	F	13	PTC	T4N1M1
10	M	67	PTC	T2N0M0
11	F	31	PTC	T2N0M0
12	F	28	PTC	T3N0M0
13	F	30	PTC	T3N1M0
14	M	32	PTC	T2N0M0
15	M	67	PTC	T2N0M0
16	F	55	PTC	T3N0M0
17	F	40	PTC	T2N0M0
18	F	47	PTC	T3N0M0
19	F	39	PTC	T3N0M0
20	F	45	PTC	T3aT0M0
21	F	33	PTC	T2N0M0
22	F	68	PTC	T2N0M0
23	F	45	PTC	T3N0M0
24	F	43	PTC	T1N0M0
25	F	77	PTC	T1N0M0

**Table 16:** Relative expression of miRNAs in papillary thyroid carcinoma (PTC) compared with matched normal thyroid tissue controls determined by qRT‐PCR

miRNA name	PTC	*N*	*P*‐value	Fold change
Upregulated				
miR‐221‐5p	10.35 ± 3.68	2.88 ± 1.15	<0.001	3.91 ± 1.36
miR‐222‐5p	7.80 ± 1.18	3.44 ± 0.73	<0.001	2.35 ± 0.52
miR‐34a‐5p	7.45 ± 1.22	2.21 ± 1.43	<0.001	2.94 ± 0.74
miR‐146b‐5p	10.39 ± 2.97	1.7 ± 0.35	<0.001	6.11 ± 1.02
miR‐21‐5p	8.03 ± 2.77	3.26 ± 0.67	<0.001	2.53 ± 0.84
miR‐31‐5p	6.52 ± 0.98	2.93 ± 0.39	<0.001	2.12 ± 0.47
Downregulated				
miR‐181‐5p	3.91 ± 1.32	7.40 ± 2.21	<0.001	2.00 ± 0.51
miR‐138‐5p	4.00 ± 1.55	7.05 ± 1.99	<0.001	1.76 ± 0.36

## Discussion

The lack of agreement among studies is a common drawback of miRNA profiling studies. Variations in experiment protocols, differences in measurement platforms, limited numbers of samples studied, and low numbers of aberrantly expressed miRNAs in comparison to relatively large total numbers of miRNAs, may render miRNA expressions levels uninterpretable. It was demonstrated that each platform is comparatively stable with respect to its own intrareproducibility. Yet, the interplatform reproducibility is relatively low among different platforms 41, 42. Furthermore, the small sample size and large numbers of features have resulted in high numbers of false negative results due to low statistical power 43.

Although the ideal method of miRNA analysis is working on the aggregated raw profiling datasets; however, it is usually unrealistic to perform this rigorous approach as the raw data are often unavailable and the interplatform result concordance is low. To overcome these obstacles, it may be a preferred solution to analyze datasets separately and thereafter aggregate the resulting miRNA list. The meta‐analysis approach was used to analyze thyroid cancer specific miRNAs obtained from independent reports. The key element of this method was searching for the most recognized miRNAs in the profiling studies. Microarray remains the most used assay for high‐throughput screening 44, 45. Due to the fact that qRT‐PCR can only detect the preselected miRNAs and the interplatform result concordance between microarray and qRT‐PCR remains low 45, we concentrated on reports that screened miRNA expression with microarray platforms.

We need to consider some factors when identifying candidate diagnostic miRNAs in thyroid cancer. In the first place, the average fold change of the candidate miRNA should be big enough to discriminate cancer samples from benign tissues. As demonstrated in Tables 2 and 3, the mean fold changes of the identified, consistently reported miRNAs from microarray platform‐based studies were all more than 2. Furthermore, we carried out a meta‐analysis in four histological subtypes of thyroid carcinoma, respectively. We observed that the meta‐signature of different subtypes of thyroid carcinoma varied considerably.

In the second place, further research on the biological functions of miRNAs are required. One miRNA may have dozens or hundreds of target genes, and one mRNA may be modulated by multiple miRNAs 7. For example, miR‐221 regulated gastric carcinoma cell proliferation by targeting phosphatase and tensin homolog deleted on chromosome ten (PTEN) 46 and could enhance growth and invasion of gastric cancer cells by targeting RECK 47. Though the interaction between miRNA and mRNA could be tumor‐specific, a deeper understanding of the molecular mechanism could contribute to advancements in clinical applications.

Thirdly, there should be adequate information about their pattern of expression in various kinds of tissues. It has been suggested that serum‐obtained miRNAs are more tissue‐specific than tumor‐specific 48, 49. In view of the fact that there are only three studies 50, 51, 52 on plasma‐based miRNAs, we included only studies that analyzed miRNA expression across thyroid cancer and normal tissues.

External experimental validation in an independent cohort of patients is often required to confirm the meta‐analysis results. We determined the expression of the eight identified miRNAs with qRT‐PCR analysis and verified that the eight miRNAs were indeed differentially expressed between PTC samples and normal thyroid tissues.

The results of the systematic review might add some information to the candidate miRNA biomarkers in thyroid carcinoma. The identified microRNAs, which are most consistently reported, may be potential diagnostic/prognostic biomarkers and therapeutic targets.

## Conflict of Interest

The authors declare that they have no competing interests.
